# Epigenetic Drivers in Pediatric Medulloblastoma

**DOI:** 10.1007/s12311-017-0899-9

**Published:** 2017-11-25

**Authors:** Martine F. Roussel, Jennifer L. Stripay

**Affiliations:** Department of Tumor Cell Biology, St. Jude Children’s Research Hospital, Memphis, TN 38105 USA

**Keywords:** Medulloblastoma, Epigenetics, MYC, MYCN, PRC2

## Abstract

Epigenetics is the process by which gene expression is regulated by events other than alterations of the genome. This includes DNA methylation, histone modifications, chromatin remodeling, microRNAs, and long non-coding RNAs. Methylation of DNA, chromatin remodeling, and histone modifications regulate the chromatin and access of transcription factors to DNA and in turn gene transcription. Alteration of chromatin is now recognized to be deregulated in many cancers. Medulloblastoma is an embryonal tumor of the cerebellum and the most common malignant brain tumor in children, that occurs only rarely in adults. Medulloblastoma is characterized by four major molecularly and histopathologically distinct groups, wingless (WNT), sonic hedgehog (SHH), group 3 (G3), and group 4 (G4), that, except for WNT, are each now subdivided in several subgroups. Gene expression array, next-generation sequencing, and methylation profiling of several hundred primary tumors by several consortia and independent groups revealed that medulloblastomas harbor a paucity of mutations most of which occur in epigenetic regulators, genetic alterations in oncogenes and tumor suppressors, in addition to copy number alterations and chromosome gains and losses. Remarkably, some tumors have no reported mutations, suggesting that some genes required for oncogenesis might be regulated by epigenetic mechanisms which are still to be uncovered and validated. This review will highlight several epigenetic regulators focusing mainly on histone modifiers identified in medulloblastoma.

## Introduction

**Fig. 1:**
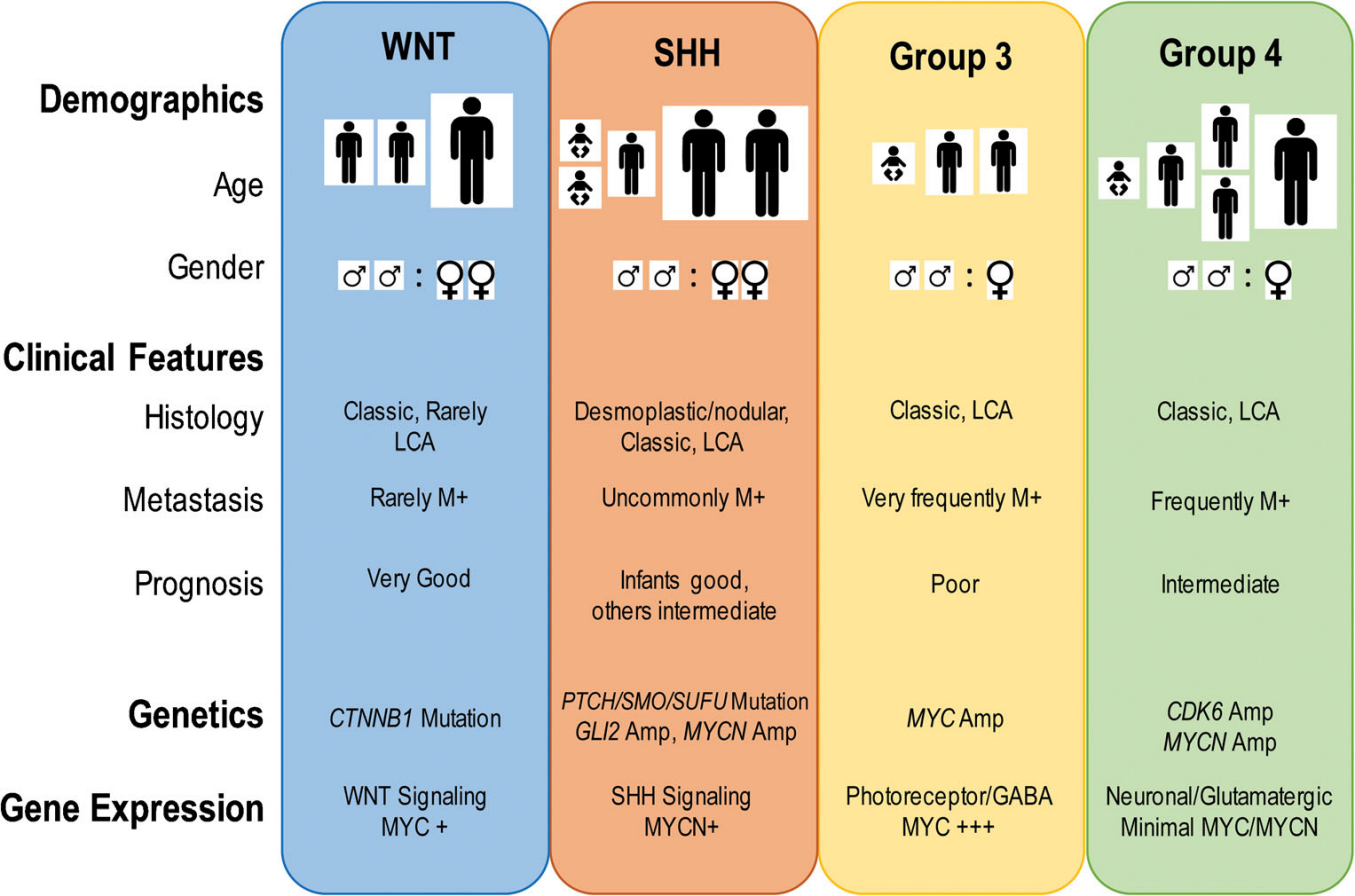
Medulloblastoma subgroups. Medulloblastoma is a heterogeneous disease composed of four moleculary and pathophysiologically distinct subgroups: wingless (WNT) and sonic hedgehog (SHH), group 3 (G3) with *MYC* overexpression, by amplification in 17% of cases, and a less well-characterized group 4 (G4). Each subgroup responds differently to current therapeutic regimen and exhibits distinct pathophysiological characteristics. Adapted from [1, 2]

Brain tumors account for the majority of solid malignancies in children, with medulloblastoma (MB) being the most malignant. MB is a heterogeneous class of embryonal cerebellar tumors that experience aberrations in pathways known to play critical roles in cerebellum development [1]. Early gene expression profiling classified MBs in four distinct molecular subtypes: two with mutations in developmental pathways, sonic hedgehog (SHH) and wingless (WNT), and two others, group 3 (G3) characterized by MYC overexpression from amplification in 17% of cases and group 4 (G4) that occurs in the highest number of patients (Fig. 1). Recent analysis of a large cohort of patients using next-generation sequencing, DNA methylation arrays, and RNA-sequencing, now subdivides each of the three subtypes SHH, G3, and G4 into additional subgroups [3–5]. Each major subtype of MB responds differently to the current therapeutic regimen that comprises tumor resection, craniospinal radiation in patients older than 3 years, and combination chemotherapy with a subset of antineoplastic agents including vincristine (antimicrotubular), cisplatin (alkylating), cyclophosphamide (alkylating), carboplatin (platinum-based), and/or lomustine (alkylating) [6, 7]. The addition of targeted therapies is currently being evaluated in clinical trials (Table 1). Current protocols have led in the past decade to increased survival rates up to ~ 70%, although patients often suffer from severe sequaela that greatly impair their quality of adult life [8]. Whereas patients with WNT MB do well with the current therapy, the prognosis for children with G3 MBs harboring *MYC* amplification or SHH MBs with *TP53* mutations and *MYCN* amplification remains poor [3]. As observed in several pediatric cancers, the mutational landscape of MBs is relatively sparse, with few recurrent genetic alterations in oncogenes and tumor suppressors, microRNAs, long non-coding RNAs (LncRNA), and regulators of DNA methylation, chromatin remodeling, and histone modifications [9]. The relative paucity of recurrent mutations across the spectrum of G3 and G4 tumors suggests that additional, non-mutated, epigenetic regulators might be implicated in shaping the chromatin signature. Besides direct alteration of genes and epigenetic regulators, gene and protein expression can be regulated by microRNAs and LncRNAs that can act as tumor suppressors or oncogenes, depending on tumor type. As a result, all these mechanisms are actively being interrogated for their role in tumorigenesis. Because epigenetic regulation is reversible, it is explored for possible therapeutic targeting. Many companies have developed compounds that target these epigenetic regulators, several of which are in clinical trials and will be highlighted throughout this discussion. This review is not meant to be inclusive of every known epigenetic regulators and their function, but rather to highlight the role of some of them in the pathogenesis of medulloblastoma.

**Table 1:** Medulloblastoma subgroups and specific experimental therapeutics. Ongoing clinical trials using targeted therapies or re-purposed drugs dictated by MB subgroup biology, genetic signature, or molecular profile

MB subgroup	Targeted therapy	ClinicalTrials.gov ID
WNT	Reduced radiation and chemotherapy dosing	NCT02724579 NCT01878617
SHH	SHH pathway inhibitors vismodegib/sonidegib	NCT01878617
Non-SHH/WNT	Pemetrexed (folate pathway inhibitor) / gemcitabine (DNA/RNA synthesis inhibitor)	NCT01878617

## Epigenetic Overview

**Fig. 2:**
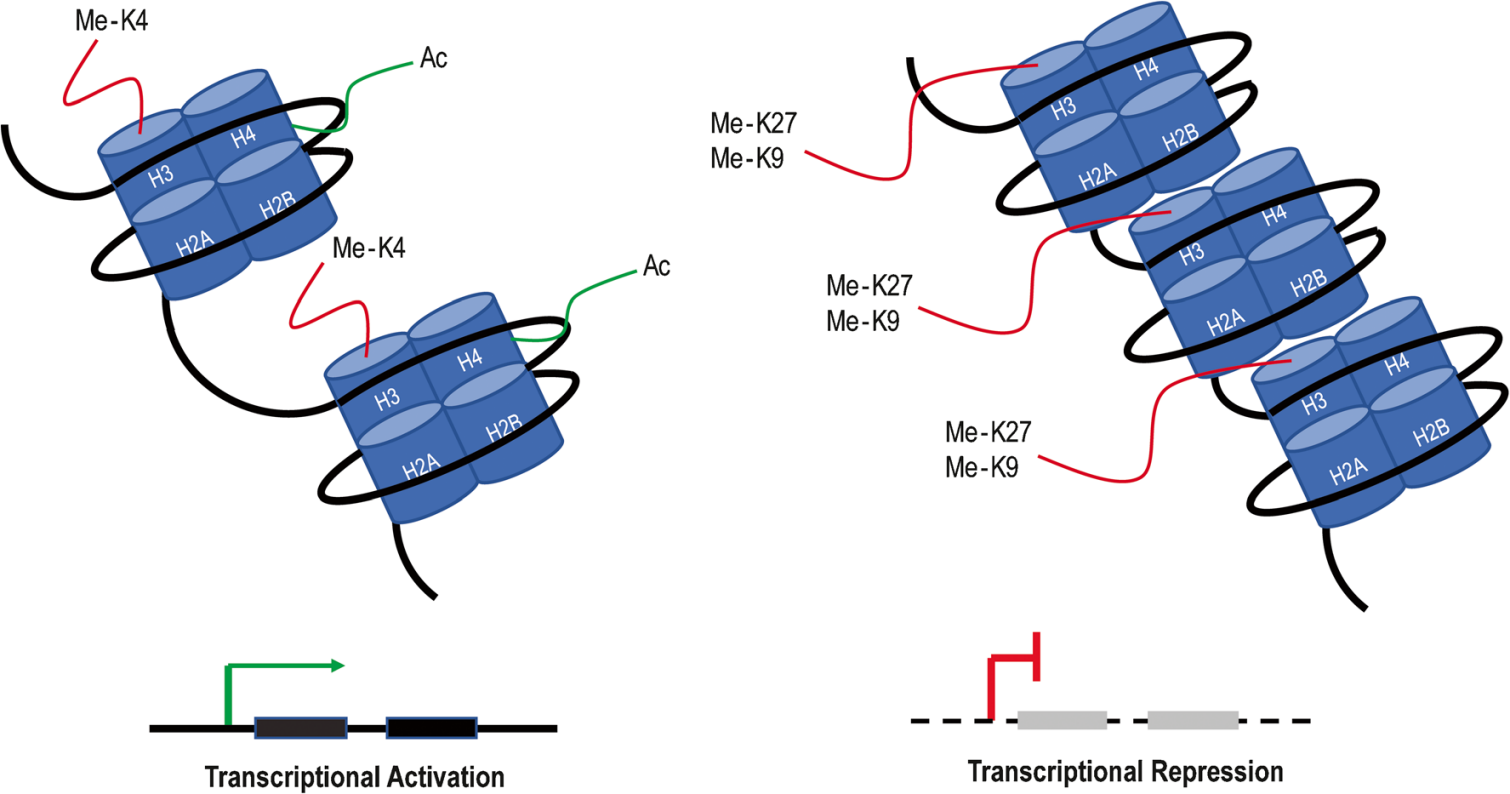
Epigenetic regulation of gene transcription by histone modifications. Histone modifications drive changes in gene expression depending on the type, extent, and location of acetylation or methylation marks. Histone acetylation (Ac) at promoters and methylation (Me) of H3K4 throughout a gene leads to activation of gene expression, while methylation of H3K27 or H3K9, particularly in promoter regions, represses transcription. Each nucleosome is composed of two copies of each histone H2A, H2B, H3, and H4

The heritable changes that do not involve perturbations in nucleotide sequences are referred to collectively as epigenetics. Epigenetic regulation is mediated by DNA methylation, histone modifications, chromatin remodeling, microRNAs (miRNA), and LncRNAs [10]. One of the most well-characterized epigenetic phenomena is methylation of DNA, which typically ensues at CpG islands in the promoter region of genes resulting in transcriptional repression. Histone modifications including acetylation, methylation, ubiquitylation, phosphorylation, and sumoylation are written, read, and erased by epigenetic modulators. Non-coding RNA (ncRNA) including miRNAs and LnRNAs regulates gene expression (typically gene silencing) at the transcriptional or post-transcriptional level. Epigenetic changes occur spatially and temporally throughout development and influence processes like cell cycle, DNA repair, and cellular differentiation [11]. Epigenetic regulation plays a significant role in cancer, often resulting in the silencing of tumor suppressor genes or activation of oncogenes (Fig. 2). In the case of mutationally “quiet” cancers, there is emerging focus on the role of epigenetics in disease pathogenesis.

## Epigenetic Mutational Landscape in Medulloblastoma

Initial studies by the Washington University/St. Jude Pediatric Cancer Genome Project (PCGP) using next-generation sequencing showed that besides amplification of *MYC*, *MYCN*, *CCND2/CYCLIN D2*, *CDK6*, and *GLI2* and mutations in *β-CATENIN*, *PTCH1*, *SUFU*, and *TP53*, mutations in epigenetic regulators account for the majority of genetic perturbations in G3 and G4 MBs (Table 2) [9, 12, 13]. The associated spectrum of altered demethylases, acetyltransferases, and nucleosome remodelers further stratifies MB subgroups [9]. The aberrant pattern of H3K4 and H3K27 histone lysine methylation occurs across subgroups in medulloblastoma [14]. Epigenetic alterations can also result in differential messenger RNA (mRNA)/miRNA expression and alternate promoter usage. Notably, many of the somatic mutations in epigenetic regulators identified in MB do not co-occur within individual tumors. As such, bourgeoning technologies like single-cell sequencing in collaboration with validation in mouse models should provide insights into which epigenetic perturbations contribute as drivers or cooperative passengers in MB.

**Table 2:** Mutations in epigenetic regulators in medulloblastoma. Check marks indicate mutations of an indicated epigenetic regulator gene in at least one tumor sample pooled from four whole genome sequencing (WGS) studies

Gene	WNT	SHH	G3	G4	*Ref.*
*ARD1A*	✓	✓	✓		[4]
*ARID2*	✓	✓			[4]
*BCOR*		✓		✓	[4, 12]
*CDH1*	✓				[9]
*CHD7*			✓	✓	[9]
*CREBBP*	✓		✓		[4, 9]
*GPS3*			✓	✓	[12]
*GSE1*		✓	✓		[4]
*KDM1A*				✓	[9]
*KDM3A*			✓		[9]
*KDM4C*			✓	✓	[9]
*KDM5A*				✓	[9]
*KDM5B*			✓		[9]
*KDM6A/UTX*			✓	✓	[4, 9, 12, 13]
*KDM7A*			✓		[9]
*LDB1*		✓			[12]
*MLL2/KMT2D*	✓	✓	✓	✓	[4, 9, 12, 13]
*MLL3/KMT2C*		✓	✓	✓	[4, 12]
*NCOR2*		✓			[12]
*PRDM6* ^a^				✓	[4]
*SMARCA4*	✓	✓	✓		[4, 9, 12, 13]
*ZMYM3*				✓	[4, 9]

^a^Enhancer hijacking drives overexpression of PRDM6

## DNA Methylation

Genome-wide DNA methylation arrays currently examine 850,000 CpG sites across CpG islands and untranslated regions (UTRs). Not only have these arrays helped confirm the four major subgroups of MBs, but they identified multiple subtypes within them [15]. These analyses suggest that DNA methylation plays a major role in the pathogenesis of MB by repressing genes to avoid cell differentiation and cell death [14]. Initial studies using bisulfite conversion of genomic DNA followed by amplification by polymerase chain reaction (PCR) revealed several tumor suppressor genes silenced by hypermethylation of CpG-dense promoters including *CDKN2A*, *HIC1*, and *RASSF1* [16, 17]. Other approaches identified repressed genes, including *PTCH1*, the negative regulator of SHH signaling, the *SFRP* family, inhibitors of the WNT signaling pathway, and the transcriptional repressor *ZIC2*, among others [18]. Some of the first epigenetic drugs proposed as anticancer therapeutics were DNA methylation inhibitors including 5-azacytidine (5-aza-CR) and 5-aza-2′-deoxycytidine (5-aza-CdR). These cytotoxic agents incorporate into DNA and trap DNA methyltransferases, preventing activity and restoring expression of tumor suppressor genes [10].

## Histone Modifiers

**Fig. 3:**
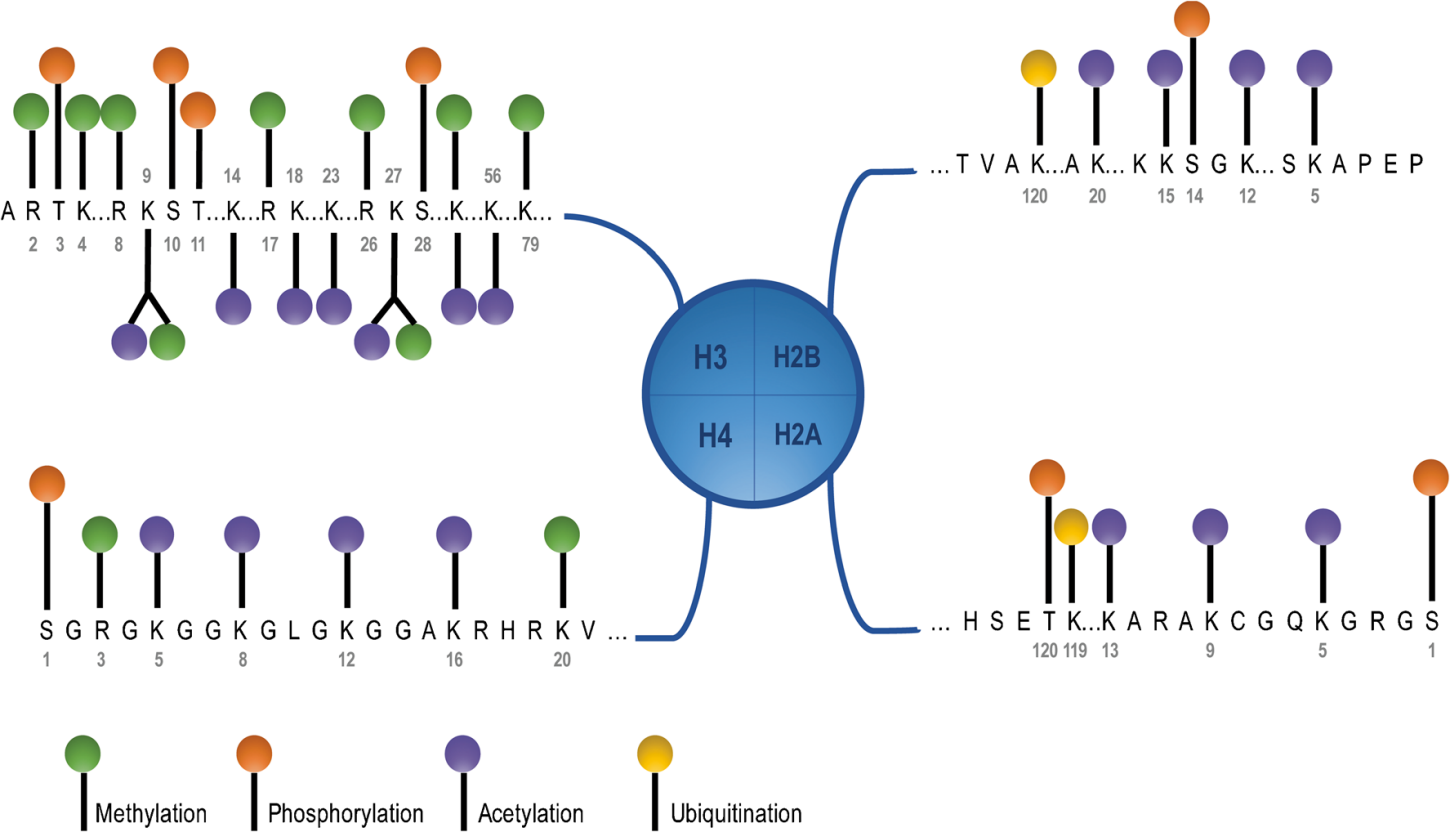
Histone tail modifications. Each of the four histone, H2A, H2B, H3, and H4, tails can be modified on several lysine (K) and arginine (R) residues by methylation (green lollipop), acetylation (purple lollipop), and ubiquitination (yellow lollipop) and on serine (S) residues by phosphorylation (orange lollipop), to regulate chromatin and DNA binding dynamics

**Fig. 4:**
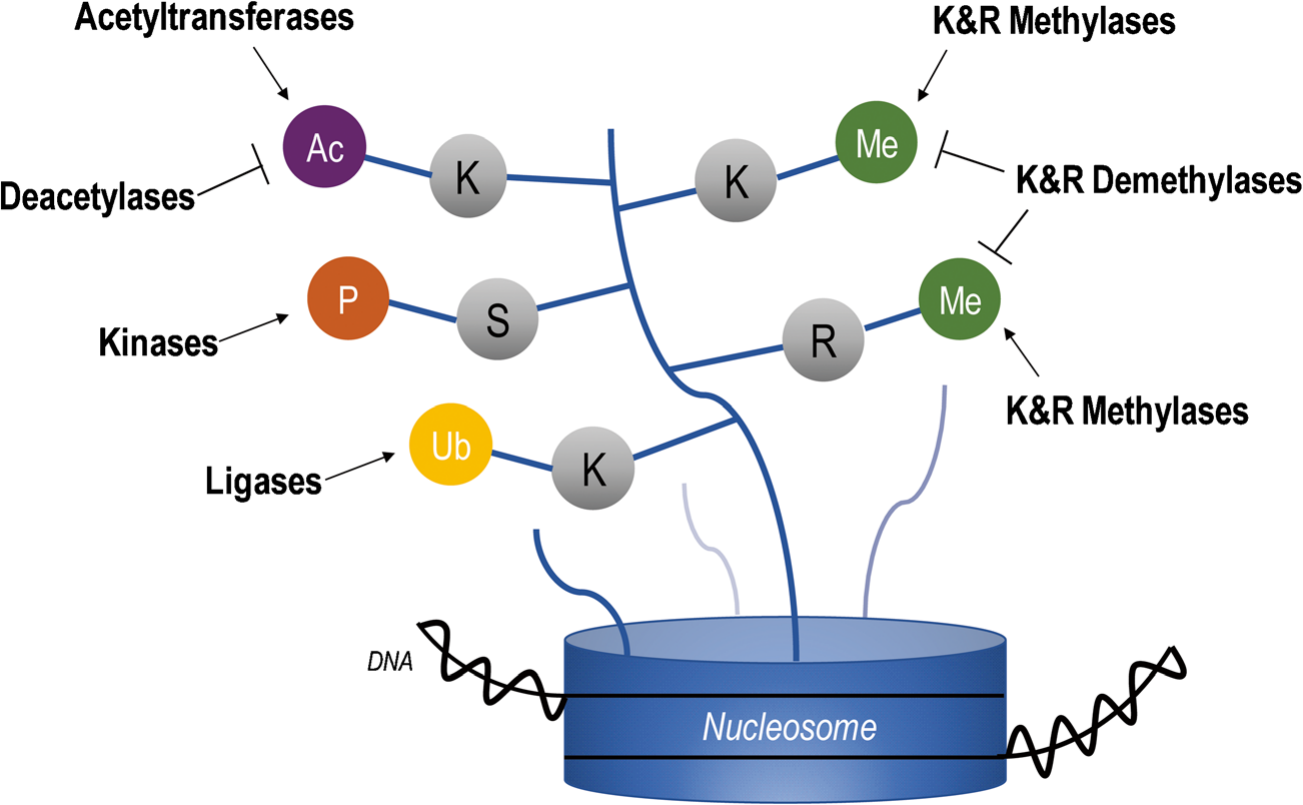
Epigenetic regulators. DNA is wrapped around the nucleosome that comprises two copies of each histone H2A, H2B, H3, and H4. Histone lysine methyltransferases and acetyl transferases (“writers”), demethylases (“erasers”), and members of the polycomb group of transcriptional repressors, PRC2 and PRC1 (“readers”), all play a role in modulating chromatin dynamics and gene expression. Mutations in these classes of epigenetic regulators have been identified across MB subgroups. Ac acetylation, P phosphorylation, Ub ubiquitination, Me methylation, K lysine, S serine, R arginine

Histones are regulated by modification of their amino-terminal tail including methylation, acetylation, phosphorylation, and ubiquitination, each of which is driven by specific enzymes within protein complexes (Fig. 3) [10]. Extensive molecular analysis of MBs identified somatic copy number aberrations and mutations in histone lysine methyltransferases (HMTs, called “writers”), demethylases (“erasers”), acetyltransferases (HAT), deacetylases (HDACs), and members of the polycomb transcriptional repressor complex, PRC2 and PRC1 (Fig. 4) [19]. Interestingly, individual histone modifiers are rarely found mutated in more than one tumor, and their frequency is relatively low from 1 to 5.8% across the four MB subgroups [9]. Inactivating mutations in *MLL2/KMT2D* and *MLL3/KMT2C*, two lysine methyltransferases that promote H3K4me2/3 associated with active chromatin and gene expression, occur in 16% of MB, with recurrence noted in SHH and G4, pointing to their role as tumor suppressors in medulloblastoma.

Histone acetyltransferases (HATs) act to relax histone-DNA interactions and promote gene transcription. Though less studied in the context of MB, HATs play a crucial role in brain development, and downregulation of the H4K16 HAT, hMOF, has been associated with poorer outcomes in MB patients [20]. Somatic alterations that target HATs are overrepresented in SHH subgroup tumors [4]. In contrast, histone deacetylases (HDACs) promote chromatin condensation and abrogate gene transcription related to cell cycle regulation and cell differentiation. Upregulation of several HDACs (HDAC5 and HDAC9, SIRT1, etc.) identified in MB contributes to aberrant cell cycle progression [21, 22]. With the goal of re-establishing normal histone acetylation patterns, HDAC inhibitors are being explored as possible therapeutics. Valproic acid, an HDAC inhibitor, induces senescence in MB cells and inhibits tumor growth by inducing expression of the cyclin-dependent kinase (CDK) inhibitory protein p21^Cip1^ [23]. Another HDAC inhibitor, trichostatin A (TSA), increases expression of the negative regulators of the WNT pathway, main drivers in this MB subgroup.

Alterations in histone demethylases are found in G3 and G4 medulloblastoma, consistent with aberrant histone methylation at H3K27 and H3K4. Trancriptional effectors of lysine demethylase (KDM) regulation include genes involved in cell cycle control, differentiation, and pluripotency. Mutations in six KDM family members have been identified, with inactivating mutations in *KDM6A/UTX* being one of the most common recurrent events in G4 MB [9, 19].

Another class of epigenetic regulators, bromodomain (BRD) and extraterminal (BET)-containing proteins, recognizes and binds acetylated histone sidechains on open chromatin and recruits the transcriptional machinery. BET/BRD proteins control MYC levels [24, 25], one of the most common driver in G3 MB [9]. A small molecule BET inhibitor (JQ1) exhibits potent antitumor activity in preclinical studies of medulloblastoma, and is currently being evaluated for chemical modification and therapeutic potential [26, 27].

## Role of the Polycomb Repressor Complex PRC2 in G3 Medulloblastoma

**Fig. 5:**
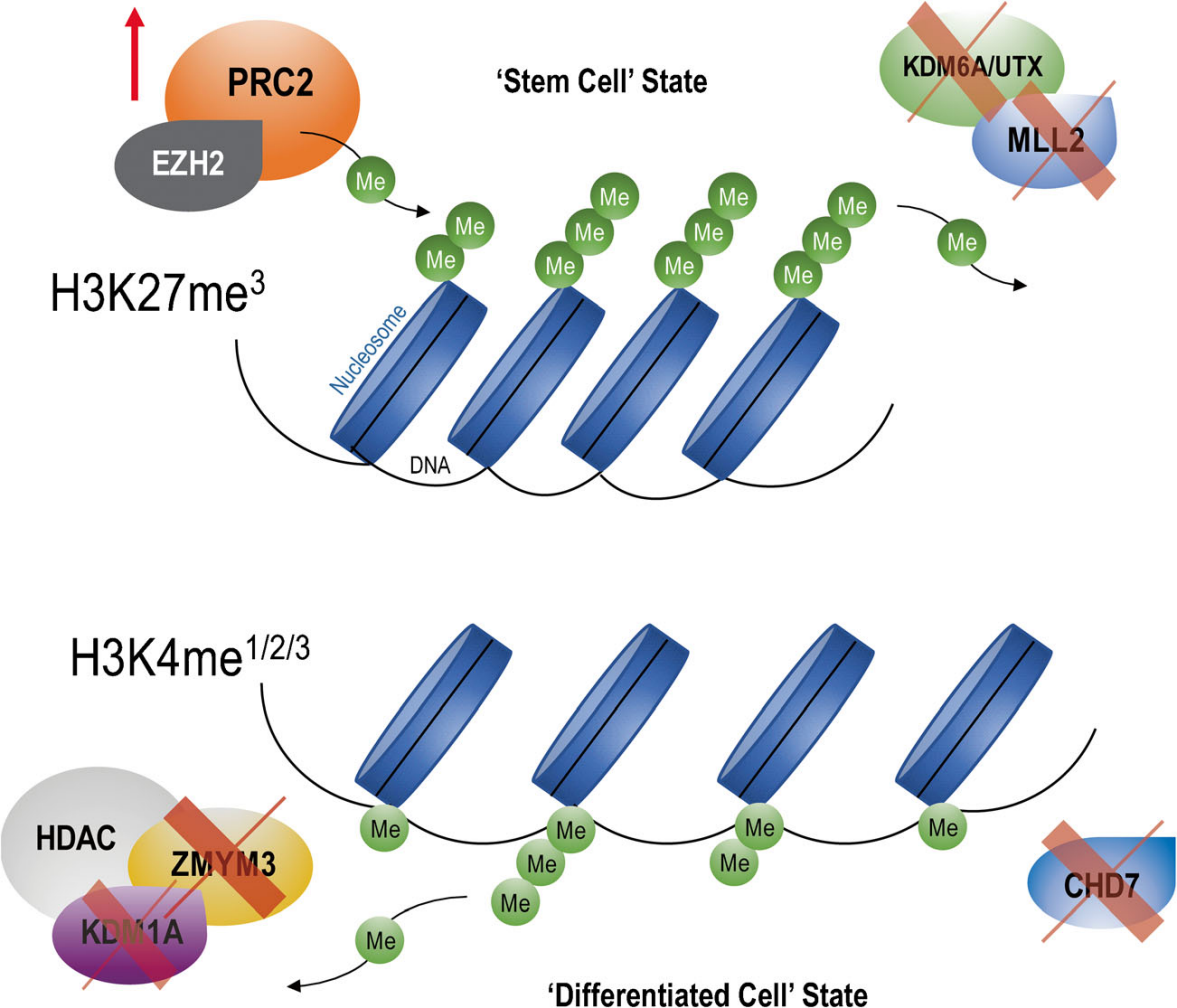
Schematic representation of bivalent chromatin domains, H3K4 and H3K27. MB retains a progenitor-like epigenetic profile by altering the balance between H3K27 and H3K4 methylation states. Aberrant “writing”, or defective “erasing,” of methyl groups of H3K27me3 (by EZH2 upregulation or *KDM6A/UTX* mutation, respectively), in addition to inactivating mutations in *MLL2/KMT2D*, *CHD7*, and *ZMYM3* that disrupt H3K4me3 transcription, establishes and maintains the stem cell state

The polycomb repressor complex PRC2 plays an important role on differentiation, maintenance of cell identity, and proliferation [28, 29]. It is miss-regulated in many cancers in which it can have oncogenic or tumor-suppressive activity depending on context. PRC2 is composed of EZH2, EED, SUZ12, JARID2, and RBAp46.48. EZH2, the catalytic partner of the polycomb repressor PRC2, is responsible for transferring methyl groups onto lysine 27 of histone 3 (H3K27me3) to contribute to chromatin compaction and transcriptional repression [30]. EZH2 is critical for normal development since its deletion in mice is embryonic lethal [31]. EZH2 is overexpressed in many cancers in which it either plays a role as an oncogene, as seen in most cases studied, or as a tumor suppressor [32, 33]. Studies by many consortia and other groups found that a subset of G3 and G4 MBs, but not SHH and WNT tumors, are characterized by high levels of EZH2 expression and H3K27me3 marks but impaired H3K4 methylation, a combination of histone marks consistent with a stem/progenitor cell-like identity that typifies G3 MBs (Fig. 5) [14]. G3 MBs can also be distinguished by their pattern of active enhancers marked by H3K27acetylation (ac) and H3K4me1 based on chromatin immunoprecipitation followed by sequencing (ChIP-seq) analysis [9, 19].

**Fig. 6:**
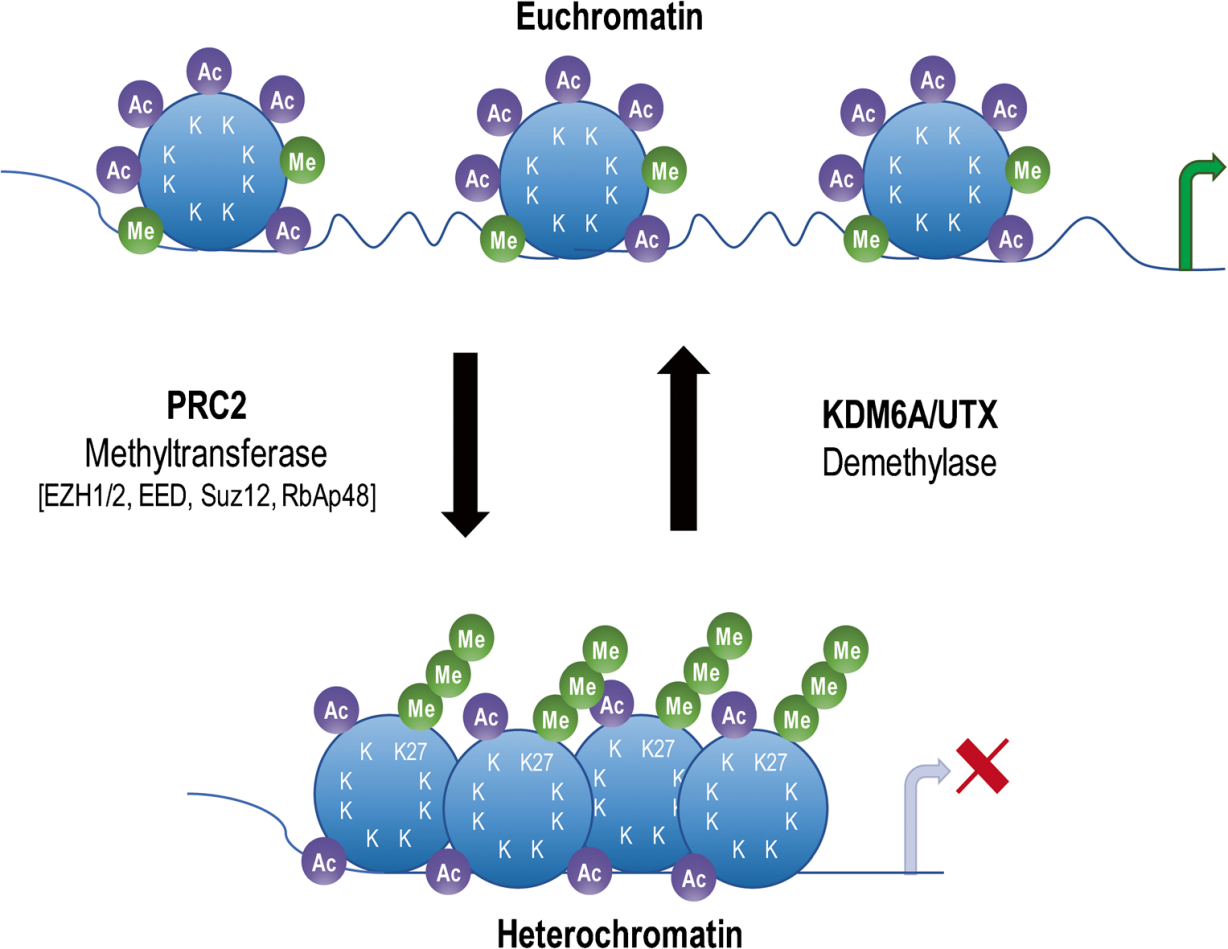
Opposite effects of EZH2 and KDM6A/UTX on transcription regulation. KDM6A/UTX (a histone demethylase) relaxes histone-DNA interactions by removing methyl groups (Me) to promote gene transcription by allowing acetylation (Ac) of histone tails (euchromatin). EZH2 (the catalytic partner of the polycomb repressor complex PRC2) transfers methyl groups to histone 3 on lysine 27 (H3K27me3) to promote chromatin condensation and abrogate gene transcription (heterochromatin). In group 3 and group 4 MBs, EZH2 expression is mutually exclusive with *KDM6A/UTX* mutations

Remarkably, *KDM6A/UTX* mutations occurring throughout the entire gene leading to complete protein loss are mutually exclusive with EZH2 overexpression, suggesting a critical role for H3K27me3 in MB tumorigenesis and potential clinical vulnerability (Fig. 6) [14]. These results suggested that EZH2 might be an oncogene, whereas KDM6A/UTX might have tumor-suppressive activity. To address whether EZH2 might have oncogenic activity, we recently deleted *EZH2* and *SUZ12* in mouse and human G3 MBs by TALEN and CRISPR-Cas9 gene editing approaches [33]. Contrary to our expectation, loss of the PRC2 complex accelerated tumor progression suggesting that the polycomb repressor PRC2 has tumor-suppressive activity. We showed that the catalytic activity of EZH2 is required for acceleration of tumor progression. Analysis of genes overexpressed in G3 MBs lacking EZH2 showed that the top 9 genes were as expected, several Hox genes including *HoxA11*, *HoxB4*, *HoxB5*, *HoxB9*, *HoxB13*, and *HoxC8* and three oncogenes, *Igfbp2*, *Erbb2*, and *Gfi1.* Interestingly, GFI1 is a transcriptional repressor which was recently reported to be overexpressed in a subset of human G3 MBs by enhancer highjacking [34]. Enforced expression of GFI1 in mouse G3 MBs accelerated tumor progression mimicking EZH2 loss, whereas GFI1 together with MYC transformed cerebellar neural progenitors and neuro-stem cells, to drive MB development, thereby bypassing a requirement for TP53 loss of function [33, 34].

Previous in vitro studies in MB cell lines using shRNA or the EZH2 inhibitor DZNEP suggested that EZH2 had oncogenic activity [35]. This apparent opposite function of EZH2 found in the two studies might be due to either the difference between the complete deletion of the protein rather than suppressing its expression or the use of cell lines passaged on plastic rather than primary tumors passaged only in mouse brain. Deletion of *EZH2* in one patient-derived xenograft of G3 MB, Icb1572, never passaged in vitro, accelerated tumor progression, consistent with data in mouse G3 MBs (Bao-Han Vo and Martine F. Roussel, unpublished results).

In accord with a context-dependent role of EZH2 as a tumor suppressor or an oncogene, targeted disruption of EZH2 by shRNA or EZH2 inhibitory drugs strongly suppressed the proliferation of other models of pediatric brain tumors. Several companies have now developed small molecules, targeting EZH2 and EED, some of which are currently in clinical trials [36]. Based on our studies using CRISPR-Cas 9 genome editing in mouse and human G3 MBs, a more thorough understanding of the consequences of tumor-specific epigenetic perturbation is warranted to avoid potentially detrimental therapeutic outcomes of EZH2 or EED inhibitory drugs in patients with medulloblastoma expressing high levels of EZH2.

## Chromatin-Remodeling Complexes and Enhancer Dynamics in Medulloblastoma

ATP-dependent chromatin remodeling complexes represent another mechanism by which histone-DNA dynamics can be altered. This class of complexes promotes the accessibility of DNA and facilitates transcription factor migration and/or histone exchange [10]. Recurrent mutations in SWI/SNF family members including SMARCA4 are identified in medulloblastoma, typically restricted to WNT and G3 subgroups [9, 37]. SWI/SNF and PRC2 complexes exhibit epigenetic antagonism, a phenomenon that has inspired the evaluation of PRC2 inhibitors in SWI/SNF mutant pediatric cancers [38]. Inactivating mutations in *ZMYM3*, a histone binding protein that typically promotes DNA integrity and contributes to the regulation of gene transcription at the H3K4me3 mark, occur exclusively in G4 tumors [39]. *ZMYM3* mutations are often found in conjunction with *KDM6A/UTX* mutations and submedian EZH2 expression, suggestive of a cooperative role and further supporting the importance of H3K27/H3K4 modulation in MB [9]. Mutations in *DDX3X*, a DEAD-box RNA helicase which plays a role in chromosomal segregation and cell cycle progression, are enriched in WNT subgroup MB and are thought to contribute directly to pathogenic β-CATENIN signaling [9].

Large-scale chromatin remodelers, including NuRD complexes, also play a role in enhancer dynamics, as contributing co-repressors or co-activators [40]. In addition to chromatin modifications in these non-coding elements driven by mutations in enhancer-associated H3K4 methyltransferases and H3K27 demethylases, *MLL3/MLL4 (KMT2C/B)* and *KDM6A/UTX*, respectively, structural rearrangements resulting in enhancer hijacking have also been described in G3 and G4 MB [34]. Changes in the enhancer landscape may contribute to inappropriate activation of oncogenes or inactivation of tumor suppressors. Recent work suggests that a putative target of enhancer hijacking, PRDM6, a presumed HMT, may serve as a prevalent driver in G4 MB [4]. Resulting “enhancer signatures” are now being investigated in MB, and other cancers, as an underlying disease mechanism and potential therapeutic vulnerability [40].

## MicroRNAs and Long Non-Coding RNAs in Medulloblastoma

The miRNAs’ and LncRNAs’ landscape in medulloblastoma has been described in the last 10 years, and some have been validated in mouse models [41]. miRNAs are short (19–25) nucleotide sequences that bind to mRNAs of protein-coding genes usually in the 3′-untranslated region of the targeted mRNA leading to translational repression or its degradation. miRNAs are generated from long transcripts encoded by the genome that are initially processed in the nucleus by the RNAse III Drosha as pre-miRNAs. Pre-miRNAs are then transported into the cytoplasm where they are cleaved by Dicer into mature miRNAs. In turn, miRNAs are incorporated into the RNA-induced silencing complex (RISC) to bind to their specific mRNAs [42]. The first miRNA reported in medulloblastoma was *miR-124*. Its expression is suppressed in MBs leading to overexpression of CDK6. CDK6, the catalytic partner of cyclin D2/CCND2 and cyclin D3/CCND3 that phosphorylates RB to drive cells through the G1 phase of the cell cycle, confers a poor prognosis in MB [43]. Another polycistronic miRNA *miR-17~92* was found to be overexpressed and to have oncogenic activity in SHH medulloblastoma [19, 44]. miRNAs can be either upregulated (*miR-17~92*) or downregulated (*miR-124*) in medulloblastoma, as in other cancers, suggesting that they could be used therapeutically. Consistent with its oncogenic activity, suppression of the *miRNA-17~92* cluster by anti-miRNAs inhibits SHH medulloblastoma development, whereas its enforced expression instead accelerates SHH tumor formation [45]. Interestingly, *miRNA-17~92* is a target of MYCN, a direct target of SHH signaling that is overexpressed by amplification in SHH MBs [46]. Many other miRNAs have been identified in each MB subgroup although the targets that they regulate have not been identified and will need validation in mouse models and patient-derived xenografts of medulloblastoma.

LncRNAs are RNAs that are transcribed in an antisense manner from any given genomic loci. Their function has been attributed to regulate integrity of the nuclear structure, regulation of gene expression, and/or post-transcriptional processing [41]. Whereas LncRNAs are aberrantly expressed in the development of the nervous system, their role and number in medulloblastoma is relatively uncharted. A recent publication identified a LncRNA (Linc-NeD125) overexpressed in G4 MBs. This LncRNA sequesters the RISC complex containing three miRNAs, miR-19a, miR-19b, and miR-106a, encoded by the *miR-17~92* cluster, leading to the de-repression of their targets, including *CDK6*, *MYCN*, *KDM6A/UTX*, and *SNCAIP*, all major drivers of G4 MB. Consistent with the role of the *miR-17~92* as an oncogene, the ectopic expression of the Linc-NeD125 suppressed G3 MB proliferation, migration, and invasion [47]. Another LncRNA that is awaiting validation is *PVT1* since it is amplified together with MYC in MBs [48]. Although few LncRNAs have been identified and validated in MBs, these recent studies suggest that they could be used therapeutically in the future. A better understanding of both microRNA and LncRNA function and dysregulation in MB may provide novel pharmacological targets.

## Conclusion

With the ultimate goal of identifying therapeutic vulnerabilities, the validation of epigenetic regulators in mouse models and patient-derived xenografts of MB should contribute to both the basic and translational understanding of MBs.

## Abbreviations

BET: Bromodomain and extraterminal domain
BRD: Bromodomain
CDK: Cyclin-dependent kinase
ChIP: Chromatin immunoprecipitation
CpG: C—phosphate—G
CRISPR: Clustered regularly interspaced short palindromic repeats
EZH2: Enhancer of zeste 2 polycomb repressive complex 2 subunit
G3: Group 3 medulloblastoma
G4: Group 4 medulloblastoma
H3Kx: Histone 3 lysine x
HAT: Histone acetyltransferase
HDAC: Histone deacetylase
KDM: Lysine demethylase
KMT: Lysine methyltransferase
LncRNA: Long non-coding RNA
MB: Medulloblastoma
miRNA: MicroRNA
ncRNA: Non-coding RNA
PRC: Polycomb repressive complex
RISC: RNA-induced silencing complex
SHH: Sonic hedgehog
shRNA: Short hairpin RNA
TALEN: Transcription activator-like effector nuclease
TSA: Trichostatin A
UTR: Untranslated region
WNT: Wingless-related integration site
